# Diagnosis and Operative Management of a Cholecystopleural Fistula Following Iatrogenic Injury

**DOI:** 10.7759/cureus.92562

**Published:** 2025-09-17

**Authors:** Angelo Federico, Marianne Chan, Adewunmi Adeyemo, John Hilu

**Affiliations:** 1 Surgery, Corewell Health, Dearborn, USA

**Keywords:** acute cholecystitis, cholecystopleural fistula, cholecystostomy tube, laparoscopic diaphragm repair, lung decortication

## Abstract

A cholecystopleural fistula is an abnormal connection between the gallbladder and the pleural space. Potential causes of this rare fistula include malignancy, trauma, iatrogenic injury, and more. Failure to recognize a cholecystopleural fistula can lead to various serious complications. We present the case of a patient who underwent an aborted percutaneous cholecystostomy tube placement for recurrent acute cholecystitis, followed by percutaneous transhepatic biliary tube placement with a clinical course that was complicated by the development of an empyema. The patient was managed operatively with a cholecystectomy and a video-assisted thoracic surgery with total lung decortication, followed by cholecystopleural fistula takedown and diaphragm repair. This case demonstrates the importance of recognizing these rare fistulas and highlights specific considerations regarding thoracic-related complications.

## Introduction

Fistulous connections between the biliary tree and the pleura are exceptionally rare. A pathologic communication between these two cavities allows for the abnormal flow of both bile and biliary pathogens from the biliary tree across the diaphragm into the pleural cavity. The most frequently isolated organisms from infected bile include *Escherichia coli* and *Klebsiella* [1]. Documented causes of these fistulas include thoracoabdominal trauma, parasitic liver disease, suppurative biliary tract obstruction, percutaneous biliary drainage, and more [2]. In Western developed countries, trauma is the most common etiology of cholecystopleural fistulas, while in developing countries obstruction secondary to liver abscesses and biliary calculus is more common [3,4]. The complications that may follow the development of these fistulas vary widely. The movement of bile into the pleural space can lead to the formation of a biliary pleural effusion with the possibility of empyema development if the effusion becomes infected. As bile is an irritant to both the lungs and diaphragm, patients can also develop respiratory distress [5]. While cholecystopleural fistulas are rare and may be confirmed with cross-sectional imaging, high clinical suspicion and early diagnosis are crucial to allow for prompt treatment to avoid these potential complications. This case report discusses the diagnosis and treatment of a patient with a cholecystopleural fistula following iatrogenic injury.

## Case presentation

The patient is a 68-year-old Caucasian male with a history of recurrent acute cholecystitis. He had previously been evaluated by multiple surgical teams and was considered a poor surgical candidate. The patient had a long-standing cardiac history, including atrial fibrillation with rapid ventricular response, coronary artery disease, and recent coronary artery bypass grafting. He was on both daily aspirin and Plavix. Because the patient was previously considered a poor surgical candidate due to his complicated past medical history, he had undergone multiple percutaneous cholecystostomy tube (PCT) placements during previous episodes of acute cholecystitis. During the most recent episode of acute cholecystitis, a PCT placement was attempted under ultrasound guidance. However, due to shadowing artifact resulting from overlying bowel, the attempt was aborted as there was no adequate window to safely complete the procedure. The patient then underwent computed tomography (CT) guided transhepatic cholecystostomy catheter placement the following day using a 10 French catheter.

**Figure 1:**
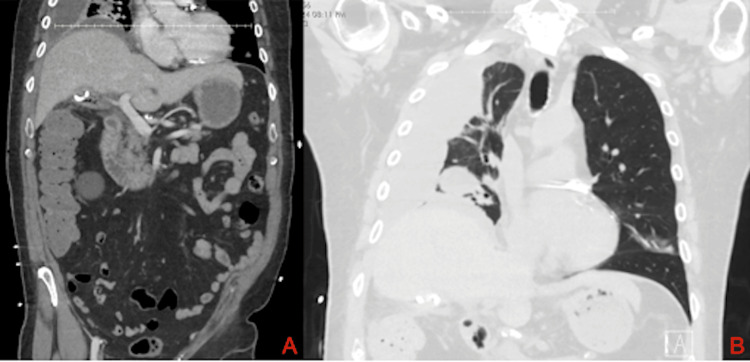
Preoperative cross-sectional imaging. (A) CT scan of the abdomen demonstrating the percutaneous cholecystostomy tube traversing the liver and curling within the contracted gallbladder with small components of gas. A partially visualized right-sided loculated pleural effusion is also seen. (B) CT of the thorax following PCT exchange again demonstrates the loculated pleural effusion, along with visualization of the PCT within the gallbladder. PCT: percutaneous cholecystostomy tube.

Two weeks after placement of the transhepatic cholecystostomy tube, the patient presented to the emergency department with concerns of abdominal pain and new-onset drainage from the insertion site. He was subsequently started on both cefepime and flagyl and underwent a CT scan of the abdomen for further evaluation. This scan demonstrated the cholecystostomy tube traversing the liver and curling within the contracted gallbladder with a small amount of gas locules, along with the interval development of a large multiloculated right-sided pleural effusion (Figure 1A). Given these findings, image-guided exchange of the cholecystostomy tube and an ultrasound-guided thoracentesis were performed the following day. Post-procedural CT of the thorax again demonstrated the loculated right-sided pleural effusion, along with the distal tip of the PCT within the gallbladder (Figure 1B). The resultant fluid analysis from the thoracentesis was exudative in nature, with cultures growing *Stenotrophomonas maltophilia*. The patient was subsequently started on oral trimethoprim/sulfamethoxazole as well given the associated antimicrobial sensitivity test. Given this Gram-negative bacterial growth, there was high clinical suspicion for contiguous spread of bacteria from the biliary tract to the pleural cavity with the most likely mechanism being secondary to iatrogenic diaphragmatic injury during the prior attempt at PCT placement.

**Figure 2:**
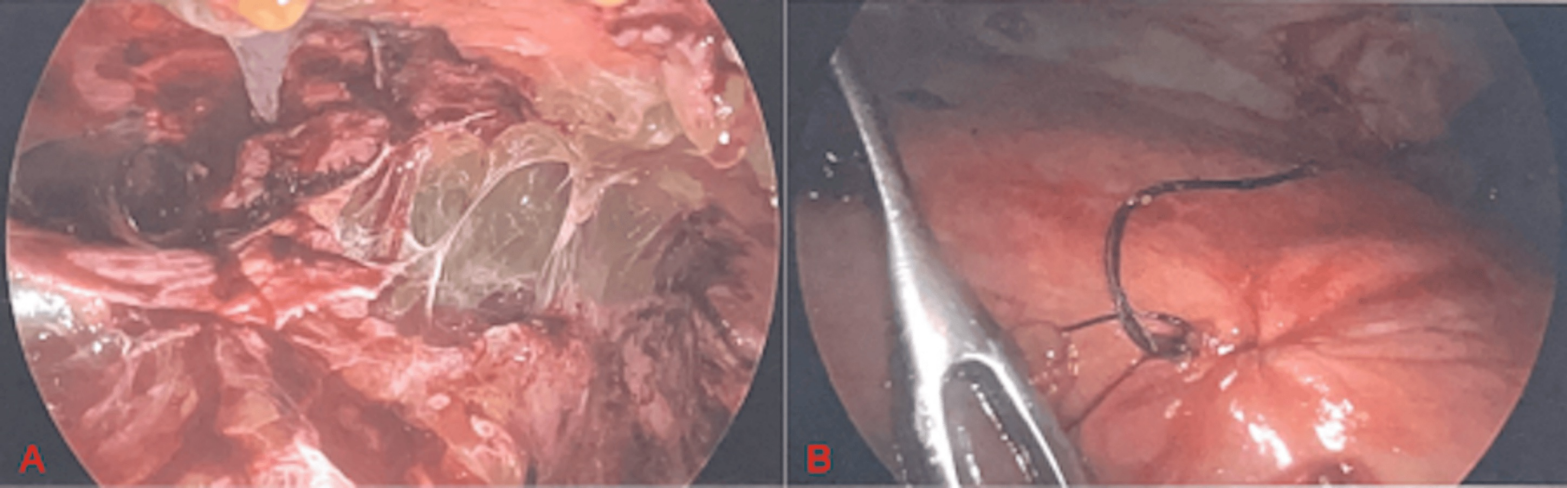
Intraoperative laparoscopic view. (A) Thick purulent exudate visualized within the right pleural cavity. (B) Visualization of the cholecystopleural fistula following closure with a 3-0 Vicryl U-stitch.

Given the patient’s history of recurrent acute cholecystitis and the newly diagnosed empyema, operative intervention was recommended. A robotic-assisted cholecystectomy was attempted, but conversion to an open procedure was necessary due to extensive dense adhesions tethering the gallbladder to both the colon and omentum. The following day, the patient underwent a right video-assisted thoracic surgery (VATS) with total decortication of the right lung. Intraoperatively, a multiloculated empyema with thick, purulent exudate was noted within the pleural space (Figure 2A). Meticulous dissection was performed to complete the decortication circumferentially around the right lung. During decortication of the anterior segment of the right middle lobe, a tear in the lung was noted and a stapled wedge resection was performed to remove this damaged portion of the lung. When attention was turned to the inferior aspect of the lung, a thick layer of rind was appreciated overlying the diaphragm. Following gentle decortication of this area, the cholecystopleural fistula was exposed and subsequently closed with a 3-0 Vicryl U-stitch (Figure 2B). The lung was then reinflated and noted to have adequate re-expansion. The patient progressed well postoperatively and was discharged the following week. Prior to discharge, a peripherally inserted central catheter (PICC) line was placed for continuation of intravenous (IV) antibiotics. The patient was discharged on a six-week course of IV ertapenem and oral trimethoprim/sulfamethoxazole. The patient followed up in the office two months after discharge and was doing well overall with complete resolution of his recurrent abdominal pain.

## Discussion

Gallbladder disease is one of the most common gastrointestinal disorders and a leading cause of hospital admissions, with approximately 800,000 cholecystectomies performed per year in the United States [6]. The complications that can follow gallbladder disease may vary widely. These complications may include diarrhea, obstruction of the bile ducts, gallstone ileus, pancreatitis, and more. While thoracic-related complications are rare following acute cholecystitis, the development of a cholecystopleural fistula is a serious complication that may occur.

Although exceedingly rare, the formation of a cholecystopleural fistula can be secondary to iatrogenic injury during cholecystostomy tube placement. This type of injury can occur if the catheter crosses the diaphragm and enters the pleural cavity during initial insertion, which is most common during a transhepatic approach [7]. The presenting symptoms can range widely from right upper quadrant abdominal pain to severe respiratory dysfunction. Patients with a fistula secondary to drainage catheter placement are at an increased risk for development of an empyema given the presence of a direct tract from the skin to the pleura, predisposing them to pleural seeding with bacterial pathogens [8]. These patients are also at an increased risk of developing an empyema from biliary pathogens given the high positive pressure within the gallbladder lumen and the negative pressure within the pleural cavity [9]. Completion of a thoracentesis with fluid analysis of a new pleural effusion in the setting of acute cholecystitis may aid in the diagnosis of a cholecystopleural fistula. In this case, pleural fluid analysis was exudative and cultures grew *Stenotrophomonas maltophilia*, which is a Gram-negative aerobic organism that is a documented cause of biliary tract infections [10]. Based on this information, we suspect the source of the empyema was from direct bacterial spread from the gallbladder into the pleural space through the cholecystopleural fistula.

Given the rarity of cholecystopleural fistulas, future research efforts should focus on educating both high-risk patient groups and healthcare professionals regarding the potential symptoms. With increased awareness, there is a possibility of both earlier diagnosis and improved treatment outcomes.

## Conclusions

Given the rarity of cholecystopleural fistulas and the limited literature available regarding both the diagnosis and subsequent treatment, misdiagnosis is not uncommon. Despite the difficulty in diagnosis, these fistulas must be identified and addressed early to avoid serious complications, as illustrated in the present case. Therefore, it is vital for clinicians to understand the diagnostic process and to maintain a high clinical suspicion for a cholecystopleural fistula, especially following a difficult cholecystostomy tube placement.
